# NeoAnalysis: a Python-based toolbox for quick electrophysiological data processing and analysis

**DOI:** 10.1186/s12938-017-0419-7

**Published:** 2017-11-13

**Authors:** Bo Zhang, Ji Dai, Tao Zhang

**Affiliations:** 1 State Key Laboratory of Brain and Cognitive Sciences, Institute of Psychology, Chinese Academy of Sciences, Beijing, 100101 China; 2 Department of Psychology, University of Chinese Academy of Sciences, Beijing, 100049 China; 3 Shenzhen Key Lab of Neuropsychiatric Modulation and Collaborative Innovation Center for Brain Science, CAS Center for Excellence in Brain Science and Intelligence Technology, the Brain Cognition and Brain Disease Institute (BCBDI), Shenzhen Institutes of Advanced Technology, Chinese Academy of Science, Shenzhen, 518055 China

**Keywords:** NeoAnalysis, Python, Electrophysiology, Analysis, Toolbox, Spike, Offline sorting, Saccade detection

## Abstract

**Background:**

In a typical electrophysiological experiment, especially one that includes studying animal behavior, the data collected normally contain spikes, local field potentials, behavioral responses and other associated data. In order to obtain informative results, the data must be analyzed simultaneously with the experimental settings. However, most open-source toolboxes currently available for data analysis were developed to handle only a portion of the data and did not take into account the sorting of experimental conditions. Additionally, these toolboxes require that the input data be in a specific format, which can be inconvenient to users. Therefore, the development of a highly integrated toolbox that can process multiple types of data regardless of input data format and perform basic analysis for general electrophysiological experiments is incredibly useful.

**Results:**

Here, we report the development of a Python based open-source toolbox, referred to as NeoAnalysis, to be used for quick electrophysiological data processing and analysis. The toolbox can import data from different data acquisition systems regardless of their formats and automatically combine different types of data into a single file with a standardized format. In cases where additional spike sorting is needed, NeoAnalysis provides a module to perform efficient offline sorting with a user-friendly interface. Then, NeoAnalysis can perform regular analog signal processing, spike train, and local field potentials analysis, behavioral response (e.g. saccade) detection and extraction, with several options available for data plotting and statistics. Particularly, it can automatically generate sorted results without requiring users to manually sort data beforehand. In addition, NeoAnalysis can organize all of the relevant data into an informative table on a trial-by-trial basis for data visualization. Finally, NeoAnalysis supports analysis at the population level.

**Conclusions:**

With the multitude of general-purpose functions provided by NeoAnalysis, users can easily obtain publication-quality figures without writing complex codes. NeoAnalysis is a powerful and valuable toolbox for users doing electrophysiological experiments.

**Electronic supplementary material:**

The online version of this article (10.1186/s12938-017-0419-7) contains supplementary material, which is available to authorized users.

## Background

Rapid advancements in electrophysiological techniques in the past few decades have enabled researchers to gather vast amounts of data from numerous neurons [1]. Meanwhile, various software tools have also been developed for data storage, exploration, and analysis [2, 3]. Electrophysiological experiments studying animal behaviors (e.g. rodents and primates) typically include a variety of data, such as the neuronal and behavioral data collected by the data acquisition system as well as any associated data indicating the animal status, the stimulus parameters, and other constraints [4]. Informative results require the processing and simultaneous analysis of all of this information; this requires analysis tools that are capable of handling neural data and performing the analysis of the data according to the experimental settings. Typically different data acquisition systems use their proprietary formats for data storage, which impedes data sharing and also makes the development of analysis tools challenging.

Currently, several commercial programs for electrophysiological data processing and analysis (e.g. Offline Sorter [5], Neuroexplorer [6]) are available. However, they are costly and often cannot satisfy the demands of the rapid developments being made in the field of electrophysiology. Subsequently, open-source software packages are constantly emerging. Some of these toolboxes, such as the Spike Train Analysis Toolkit [7], the FIND [8] and the Chronux [9], are very popular. However, these toolboxes are developed based on MATLAB, which is not a free program, and may not be affordable for laboratories with a limited budget. Therefore, it would be better to develop a toolbox to process electrophysiological data based on open-source software. In this study, we propose using a programming language that has been widely used in scientific computing and is compatible with all major operating systems including Windows, Linux, and Mac OS: Python, a freely available program with plenty of existing open-source packages. Additionally, using certain packages like pandas [10], Python is capable of integrating different types of data, including numbers, strings, and lists into one data table regardless of the length of each element. This flexibility and compatibility make Python very suitable for handling the complex electrophysiological data, which contain multiple types of data.

In fact, several Python-based toolboxes have already been developed to process and analyze specific portions of the electrophysiological data. For example, Neo is a useful package aiming to standardize electrophysiological data and solve the data format problem [11]; Klusters can be used to perform offline spike sorting [12]; Spyke Viewer can be used to analyze spike trains [13]; and Elephant can be used to analyze spike trains and time series data, including local field potentials (LFP) [14]. However, in practice, using a variety of different toolboxes during data analysis is inconvenient and inefficient. Among them, the Klusters still requires the input data be formatted in specific ways, making it difficult to use. More important, although these toolboxes provide basic plotting functions, they do not take into account the sorting of experimental conditions. To plot the result of a specific condition, users have to manually sort the data before using these toolboxes. One toolbox that integrates all of these functions, including spike sorting, spike train and LFP analysis with condition sorting, and supports all data formats for the major data acquisition systems, will facilitate the efficient analysis of electrophysiological data. Recently, Garcia et al. [4] proposed a similar framework for data storage and analysis called OpenElectrophy. However, they did not provide specific analysis and statistic functions. Even in the proposed data frame, it is difficult to visualize data and the user interaction requires further improvement. In particular, this toolbox does not take into account the detection of saccades, which are important behavioral indicators for some experiments [15, 16]. In this study, we developed a toolbox called NeoAnalysis, which overcomes the shortages of other packages and facilitates efficient analysis.

## Implementation

### Design principles

**Fig. 1:**
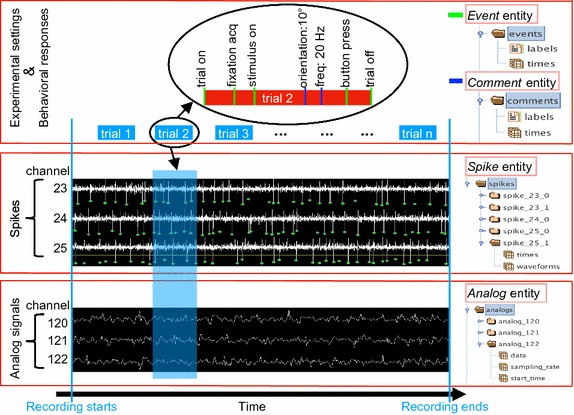
The principle for data standardization in a typical electrophysiological experiment. The experiment normally runs in a trial-by-trial manner, so the data collected must include the experimental settings defining the conditions in each trial and the behavioral and neuronal responses. These data can be divided into *Event*, *Comment*, *Spike* and *Analog* entities. The *Event* entity represents the occurrence of specific events, such as a stimulus is turned on or a button is pressed (green bar in the top panel). The *Comment* entity contains information that defines the experimental settings, such as the orientation of the stimulus in each trial (blue bar in the top panel). The Spike entity records the action potentials (middle panel), and the *Analog* entity records analog signals including local field potential (bottom panel). For a given data set, we convert the data into these four entities regardless of their original format and save in a standardized format for future use. The right side shows how these entities are organized in the output file

NeoAnalysis aims to provide an out-of-the-box tool for users to process and analyze different types of data obtained during a typical electrophysiological experiment. To this end and overcome the aforementioned shortages of existing toolboxes, we develop NeoAnalysis following the principles described below:

The first concern is the data format problem. Currently, different data acquisition systems save the recorded data in their proprietary file formats. We want NeoAnalysis to support most formats for the major commercial systems including Blackrock (Blackrock Microsystems LLC, Utah, USA), Plexon (Plexon Inc., Dallas, TX, USA), and TDT (Tucker-David Technologies; Alachua, FL, USA). Therefore, it is necessary to convert the input data to a standardized format, which will ease the subsequent work in scripting analysis programs. As we know, a typical electrophysiological experiment normally collects spikes, analog signals (e.g. LFP), experimental settings, and behavioral responses (Fig. 1). The solution is to divide these data into four basic entities regardless of their original formats: *Spike*, *Analog*, *Event*, and *Comment*.

- A *Spike* entity contains the time points at which the action potentials occur, as well as their waveforms and unit classification.
- An *Analog* entity contains the continuous data that was recorded at a given sampling frequency, such as LFP.
- An *Event* entity contains the time points and the labels defining the occurrence of specific events, such as the onset of a stimulus or the occurrence of a button press.
- A *Comment* entity contains the time points and the labels that define the experimental settings, such as the orientation of the stimulus in each trial.

With the help of Neo [11], which is an open source package aiming to standardize electrophysiological data, the input data can be easily imported to generate the *Spike*, *Analog* and *Event* entities. However, the obtained *Event* entity (*Neo_Event*) actually includes both events and comments according to our definition. Given comments are required for condition sorting during data analysis, mixing these two entities will make this procedure difficult. So we extend the Neo package in our program to further divide the *Neo_Events* to the real *Event* entity and *Comment* entity according to our definition. Then, we save these entities in a standardized format. Examples of how these entities are organized in the output file are shown on the right side of Fig. 1. This procedure substantially eliminates the limitation due to data format and enhances the compatibility of this toolbox.

**Fig. 2:**
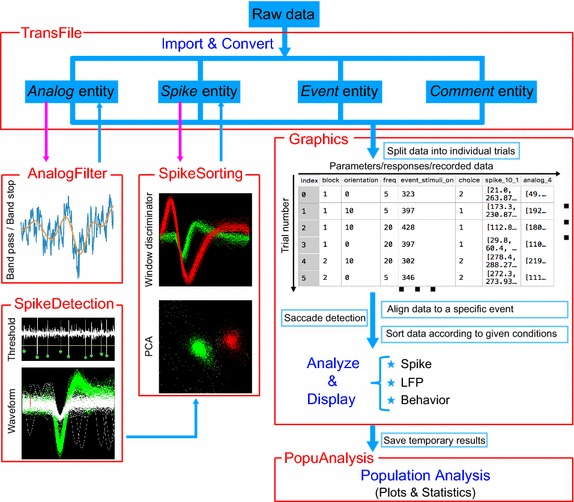
The six major modules of NeoAnalysis and their functions. Each panel represents one module, and arrows indicate the general workflow. The TransFile module is used to import data from any of the supported formats and extract the data to *Analog*, *Spike*, *Event* and *Comment* entities, and then convert to a standardized format (HDF5). The AnalogFilter module is used to filter analog signals. The SpikeDetection module can detect spikes from the analog signal and then feed to the SpikeSorting module, which resorts spikes offline. The graphics module first organizes all of the relevant data into a data table on a trial-by-trial basis for data visualization, and then provides functions to analyze the data and display the results. This module supports data computation and saccade detection in addition to common analyses of spikes, local field potentials, and other behaviors. The last module, PopuAnalysis, can retrieve the saved result of each single session and perform population analysis

Secondly, to ensure the toolbox can handle the entire workflow for electrophysiological data analysis, we design six modules for users to import and convert data, detect spikes, perform offline spike sorting, filter analog signal, analyze single session data and population data (Fig. 2).

#### TransFile

The TansFile module assists users to convert the raw data of any supported format to an HDF5 file using the preceding principle. The HDF5 format is used because it is a highly efficient format for data I/O, especially for data of a large volume and a complex structure. In addition, it is a unified format that can be used by different operating systems and programming languages [17].

#### SpikeDetection

The SpikeDetection module is used to detect spikes from the raw signals. Given most data acquisition systems support online spike detection, this module will not be used for most users. In the case when non-standard recording systems are used and additional spike detection procedure is required, we designed this module for users to detect spikes with a graphic user interface (GUI). In addition to manually setting the threshold and selecting waveform [18], this module supports automated spike detection based on a widely used unsupervised algorithm proposed by Quiroga et al. in 2004 [19].

#### SpikeSorting

The SpikeSorting module is for resorting spikes with a GUI. In the current design, it supports both automated and manual sorting. The algorithm for automated sorting is an unsupervised method based on wavelet analysis and superparamagnetic clustering [19, 20]. In cases when users are not satisfied with the result of automated sorting, we provide additional manual options: the window discriminator and the principal component analysis (PCA) discriminator. Users can either use the line widget to select waveforms or the polygon widget to select data points for re-sorting. We also provide a 3D view of the first three principal components. This 3D space can be rotated, zoomed in, and zoomed out to reveal the distribution density of the data set in this space with appropriate transparency.

#### AnalogFilter

The AnalogFilter is used to filter the *Analog* data using common filters including band-pass, band-stop, low-pass, and high-pass filters. For each of these filters, users can select the filter method from the available list including Chebyshev I, Chebyshev II, Cauer/Elliptic, and Bessel/Thomson. In addition, users can define their own filters. The current design provides a GUI window for users to perform filtering. Alternatively, users can also use command scripts.

#### Graphics

The graphics is a module used for data visualization and analysis. In our design, this module first groups data into a table on a trial-by-trial basis according to experimental conditions, and then allows users to perform analyses such as plotting PSTH. In the data table, a row represents a trial, and a column represents a specific type of data, such as stimulus onset time, offset time, reaction time, spike, and LFP. There is also an available option to customize the table to include only selected trials using specific settings (specified by the parameter ‘*limit*’). This is very useful when you only want to analyze a portion of the data. For the data in the table, the graphics module can do more than just plot the whole group of data; it can also sort the data according to the given conditions (specified by the parameter ‘*sort_by*’). The sorting function here supports up to two levels of conditioning. For example, one experiment studies the difference in orientation tuning using grating stimuli of different spatial frequencies. Suppose there are two spatial frequencies and eight orientations, then the sorting function can classify the results into two major categories, each category containing eight conditions. For spike train analysis, the graphics module provides functions to draw raster, PSTH, and the accumulated spike counts; for analog data (e.g. LFP), the graphics module can draw the average signals and perform spectrum analysis or a time–frequency analysis; for behavioral responses (e.g. choices, reaction times), the graphics module provides several general-purpose functions to analyze scalar values. Additionally, the graphics module provides functions to calibrate eye position, to detect saccades, and to extract saccade information. For all of these analyses, the results can be stored in a workspace for further analysis (e.g. population analysis). The graphics module does not provide any GUI for the aforementioned functions. Instead, users need to type their commands through the Python command window in order to run a specific function. All of the functions provided here have several opening settings, and users can easily change these parameters to obtain the required results. Therefore, the commands are more flexible in terms of analyzing complex electrophysiological data.

#### PopuAnalysis

In order to analyze the electrophysiological data at the population level, we designed the PopuAnalysis module. This module uses the results stored in the workspace obtained from analyzing single session data. For the spike train analysis, this module can plot the mean PSTHs for different conditions across a population of neurons. For analog data, this module can plot the mean signal, the power spectrum, and the time–frequency spectrum for different conditions across the population. For behavioral data (e.g. reaction time), this module can plot the mean values with error bars across the population. In addition, this module provides several commonly used statistical functions including descriptive statistics, t test, and ANOVA.

### Features and capabilities

Following the design principles, NeoAnalysis has the following features:

- NeoAnalysis supports most data formats from the major commercial data acquisition systems through combining the Neo package [a powerful open-source module for data input/output (I/O)] [11].
- NeoAnalysis provides user-friendly GUIs and data viewing through integrating the open-source module PyQtGraph [21]. The PyQtGraph is a Python based graphics and GUI library, which uses less memory and performs much more efficiently than simply using the embedded graphic library ‘matplotlib’ [22]. Furthermore, NeoAnalysis puts a lot of emphasis on user interaction design. In particular, it provides several easy-to-use widgets for offline spike sorting.
- NeoAnalysis groups all of the experimental information, including the recorded signals, behavioral responses, and the results of preprocessing into a table on a trial-by-trial basis. This informative table can be easily displayed and can be further sorted according to given conditions (e.g. experimental conditions). Furthermore, NeoAnalysis provides many other functions to manipulate the table and run the further analysis.
- NeoAnalysis provides a complete workflow for electrophysiological data analysis, which covers data standardizing, data preprocessing, single unit analysis, data storage, and population data analysis. Throughout the entire data analysis process, users do not have to switch between different programs and toolboxes. More important, NeoAnalysis takes into account the experimental conditions and supports analyzing with automatic condition sorting. Therefore, users can obtain sorted results by simply specifying parameters in the commands without writing additional scripts.
- NeoAnalysis is capable of processing eye movement information, including calibrating eye position and detecting saccades. During experiments, when recording eye movement trajectories, it is essential to detect the occurrence of saccades and to extract the relevant information. Previous open-source toolboxes generally do not provide such functions.
- Due to the incompatibilities between Python 2.7 and Python 3.5, NeoAnalysis provides two slightly different versions for these two releases.

## Results

### Procedures of analysis using NeoAnalysis

After successfully installing NeoAnalysis^1^, users can use the toolbox following the procedures depicted in Fig. 2. A step-by-step tutorial can be found in the user manual.^2^ In brief, users first import the raw data of any supported format and convert to HDF5 format. Then users can perform spike detection (see Additional file 1), spike sorting and/or signal filtering on the converted data. Next, if the experiment includes data regarding eye movement, users can perform saccade detection and extraction. Otherwise, users can begin to analyze spike trains, LFPs, and other behavioral data using the corresponding plotting functions. The results of each analysis session can be saved for future use. If users want to analyze the data for a population of neurons, NeoAnalysis can retrieve the saved workspace and perform analysis and statistics across the data gathered for an entire population.

### Spike sorting

**Fig. 3:**
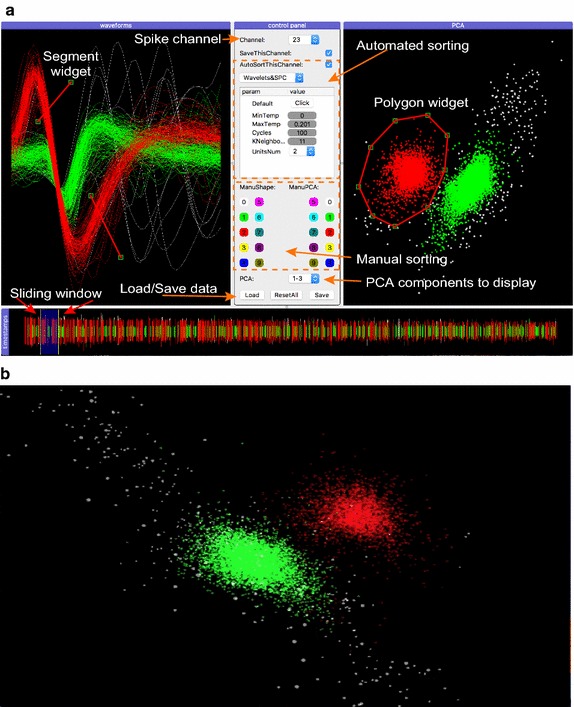
The graphic user interfaces for offline spike sorting. **a** The main interface, in which the center shows the control panel for major operations; the bottom panel shows all spikes in the selected channel with a sliding window to select a portion of spikes; the left panel shows the waveforms of the selected spikes, and the right panel shows the principal components of all spikes in the selected channel. Users can check the AutoSortThisChannel box to start automated sorting. In addition, users can use the segment widget (two red lines with square ends) to select waveforms or use the polygon widget (red polygon with square nodes) to select data points for re-sorting. **b** A 3D view to display the first three principal components of all spikes in the selected channel

Offline spike sorting can be done using the SpikeSorting module. The following codes are used in order to start the module with a 3D view: 

An interface with several buttons and panels is then displayed. Users can load data from a specific location by clicking the *Load* button at the bottom of the control panel. All of the spike channels will be shown in the drop-down box labeled as ‘Channel’, and users can select the channel of interest to begin the sorting process. All of the spikes recorded in the selected channel are shown in the bottom panel (labeled as ‘timestamps’). A sliding window is provided so that a portion of the spikes can be selected to display their waveforms, which are shown in the left panel (labeled as ‘waveforms’). The right panel shows the principal components of all spikes in the selected channel (labeled as ‘PCA’, Fig. 3a). Users can check the ‘AutoSortThisChannel’ box to start automated sorting using the wavelet analysis and superparamagnetic clustering method [19, 20]. The parameters displayed below have been set to be optimal based on a previous study [19]. Generally, this function generates satisfying results without adjusting these parameters. Though users can adjust the *UnitsNum* to define the number of sorted units, this actually does not change the result of the major units but only assign those units of minority spikes to the unsorted one (unit 0). In cases when users are not satisfied with the automated sorting, we provide the option to sort manually. Users can choose either the window discriminator or the PCA discriminator to perform the sorting. When using the window discriminator, users can use the segment widget (two red lines with square ends, which can be moved, stretched, shortened, and oriented) to select spike waveforms in the left panel. When using the PCA discriminator, a polygon widget (red polygon with square nodes, which can be moved, reshaped, and edges can be added or removed) is provided to select spike principal components in the right panel. The selected spikes can then be assigned to unit 1–unit 9 (unit 0 means unsorted). It is important to note that any re-sorting done using either discriminator will be simultaneously displayed in both panels. In the meantime, this module provides a 3D view of the first three principal components of all the spikes in the selected channel (Fig. 3b). Even though no other operation is allowed, it provides users an overview of the data and helps users verify the selection using the PCA discriminator. After users are satisfied with the sorting results, they can click the *Save* button to save the data. Otherwise, they can click the *ResetAll* button to start over. An additional movie shows this procedure in more detail (see Additional file 2).

### Single unit analysis

The graphics module of NeoAnalysis provides users several useful functions to perform the basic analysis. A remarkable feature of these functions is that they are all equipped with a powerful ‘*sort_by*’ option, which allows users to obtain results according to the experimental conditions (see “Design principles”). The graphics module first provides users a data table that includes all of the experimental information and the recorded signals on a trial-by-trial basis. Then, through the use of the ‘*sort_by*’ option in combination with other settings, users can obtain the required results without having to write complex codes. The following command lines illustrate how the graphics module computes PSTH, plots raster and calculates spike counts.

In the Python console window, run the following codes: 

In line 1, the graphics module from NeoAnalysis is imported for single unit analysis. Line 3 defines the path and the filename of the data. Line 4 initiates the graphics class by setting the parameters *filename*, *trial_start_mark* and *comment_expr*. The *trial_start_mark* is the marker representing the start of a trial, which is used to separate the raw data into different trials. The *comment_expr* tells the program how the experimental conditions and parameters are stored in the data. In this example data, the experimental condition (here is ‘patch_direction’) and the setting of each trial (here is a value in degree) are stored together as a *comment* entity with a semicolon in between (i.e. ‘patch_direction:degree’). By setting the *comment_expr* as ‘key:value’, the program decodes the key as ‘patch_direction’, and the value for a particular trial is the degree of that trial. This option provides users the flexibility to store their experimental parameters. After this step, all data are reorganized into an informative data table on a trial-by-trial basis, which can be displayed using the code in line 5. A portion of the table is shown in the graphics panel of Fig. 2.

Considering that experimental conditions are stored as ‘string’ in the data, converting them to ‘numeric’ will make the sorting faster during conditioning, as the data are sorted by their logical orders. This is done using the code in line 6.

**Fig. 4:**
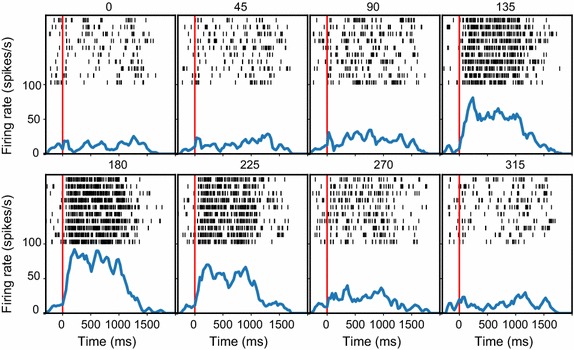
The raster plots for the sample data generated by the graphics module. Each panel represents the response to one condition defined by the setting *sort_by* (here is the drifting direction of the random dot patch)

Raster with accumulated PSTH can be plotted using the function in line 7. Most parameters, including *bin_size*, *overlap*, *Mean*, *Sigma*, *filter_nan*, and *fig_column* have default values, which means that users do not necessarily have to input these parameters if they do not have particular requirements. Users do need to define the parameters *channel*, *sort_by*, *align_to*, *pre_time*, and *post_time*. The *channel* parameter defines the spike channel and the unit order, in case there are multiple units recorded. The *sort_by* parameter defines which experimental conditions are used to sort the data. The *align_to* parameter defines which event marker is used to align the data. In this example, the event marker ‘event_64721’ represents the onset time of the visual stimuli. The *pre_time* and *post_time* parameters represent the time range (relative to the *align_to* parameter) selected for the analysis. The *bin_size* and *overlap* parameters represent the bin width for computing the PSTH and the overlap between two adjacent bins. The *Mean* and *Sigma* define the Gaussian kernel for data smoothing. The output of line 7 is shown in Fig. 4, which shows the smoothed PSTH at the bottom and the raster at the top of each panel. Notably, this function does not just plot a figure, it also allows for plotting the results according to the required experimental conditions.

**Fig. 5:**
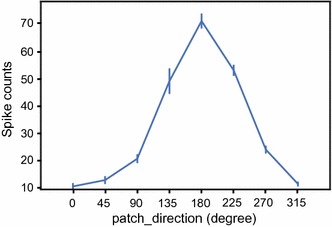
The line plot of the spike counts for the sample data. Each point represents the spike count within a given period for one condition defined by the user (same as Fig. 4)

The command in the line 8 plots the spike counts during the period defined by the parameter *timebin*. Other parameters use the same convention as in line 7. The output of this command is shown in Fig. 5, which shows the direction tuning of this example neuron.

### Spectrum analysis

**Fig. 6:**
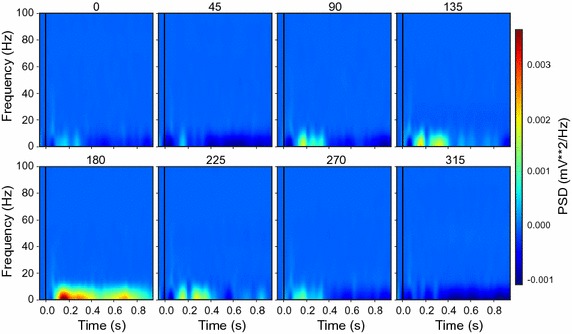
The time–frequency analysis for the sample data using the graphics module. The function analyzes the power density over the low-frequency domain (< 100 Hz) during a given period. Each panel represents the result for one condition (same as Fig. 4)

A common analysis for LFP is to plot the spectrogram. The graphics module provides several functions to perform the spectrum analysis using the periodogram method [23]. For example, the function below plots the time–frequency spectrum of LFP for the low frequency domain (< 100 Hz): 

This function sorts the data in channel ‘analog_26’ using the *patch_direction* parameter, and the time window defined by *pre_time* and *post_time*. Setting the *color_bar* to be ‘True’ turns on the scale bar. By default, the function uses a ‘hann’ window to calculate the density across the time–frequency domain. Users can refer to the manual for more details about the available options. The result is shown in Fig. 6.

### Saccade detection

**Fig. 7:**
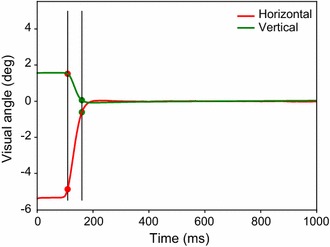
An example of the saccade detection using the graphics module. The red and green lines represent the horizontal and vertical eye position, respectively. The black vertical lines indicate the start and end time, and the red and green spots indicate the start and end position of the detected saccade, respectively

NeoAnalysis provides a function called *find_saccade* to detect saccades. The algorithm for saccade detection in this function is based on setting thresholds for eye movement speed, duration, and distance [24]. These parameters have already been set to optimal values, according to our experience; however, users can reset these parameters if the default settings do not satisfy their needs. The results of saccade detection contain information regarding when and where a saccade starts and ends, as well as the amplitude of the saccade. This information is also added to the aforementioned data table that contains all of the experimental settings and recorded signals. In addition, NeoAnalysis provides another function called *choose_saccade*, which can be used to select saccades during a given period of time and/or within a certain range of amplitude. An example of saccade detection is illustrated in Fig. 7, in which the black vertical lines indicate the start and end times, and the red and green spots indicate the start and end positions of the detected saccade, respectively.

### Data analysis at population level

The results obtained from the analysis discussed above can be stored in a workspace for each recording session. NeoAnalysis then provides a module, named PopuAnalysis, to analyze the population data across all sessions. In the following example, we illustrate how to use this module to analyze behavioral and electrophysiological data at the population level using a simulated workspace named ‘sample_workspace.h5’. 

**Fig. 8:**
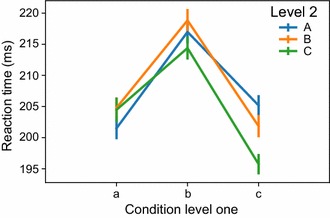
The behavioral data analysis at the population level for the sample workspace. This example analyzes the reaction time for three level 1 conditions (a, b, c) under three level 2 conditions (A, B, C). Error bar indicates SEM

Using the codes above, first the workspace in the data folder is loaded (line 1-3), and then the mean reaction time is computed for the different experimental conditions with line plot displays (line 4). The parameter *store_key* in line 4 defines which data will be analyzed in the workspace, and the parameter *conditions* defines the conditions for data sorting. In this example, there are two levels of conditions, with each level containing three factors (‘a’, ‘b’, ‘c’ and ‘A’, ‘B’, ‘C’ for level 1 and level 2, respectively). The result of this analysis is shown in Fig. 8.

**Fig. 9:**
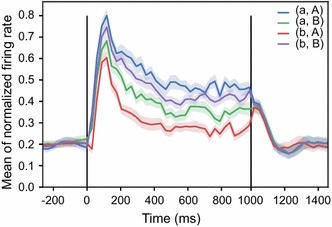
The mean firing rate of a population of neurons. This example shows the mean of the normalized firing rate for four conditions across the population in the sample workspace. Different colors indicate the different conditions. Shading indicates 68% confidence interval

For spike train analysis at the population level, the function *plot_spike* is used: 

The command line above compares the neuronal activities among four experimental conditions: *(*‘*a*’, ‘*A*’*)*, *(*‘*a*’, ‘*B*’*)*, *(*‘*b*’, ‘*A*’*) and (*‘*b*’, ‘*B*’*)*. The parameter *store_key* defines the data to be analyzed. If the parameter *normalize* is set to be *True*, the neuronal activities from different neurons will be normalized before calculating the mean responses. The *fig_mark* denotes where to put the vertical reference lines to indicate specific events (e.g. stimulus onset). The *error_style* sets the error bar style in the figure and *ci* sets the confidence interval. The result of this command is shown in Fig. 9.

## Discussion

### Comparison with other toolboxes

We have illustrated the implementation of the NeoAnalysis toolbox, which we have shown to be quite powerful and efficient as compared to other open-source packages. The NeoAnalysis covers the whole workflow for electrophysiological data analysis, while the Klusters [12] and the Spyke Viewer [13] can only perform a portion of the analysis. More important, NeoAnalysis can easily generate sorted results based on the given conditions (up to two levels) without writing additional scripts, while other toolboxes require users to manually sort data beforehand. Therefore, these ready for use functions provided by NeoAnalysis reduce the requirement for users’ scripting abilities and substantially improve the analysis efficiency.

There is another open-source toolbox called OpenElectrophy [4], which has a similar scope as NeoAnalysis but does not provide specific analysis and statistic functions. OpenElectrophy uses MySQL, an open-source database, for data storage. Interacting with the data in the database is a challenge for users who are not familiar with structured query language (SQL). Furthermore, the data in the database is stored as tables and the relation between different tables is complex. Additionally, despite OpenElectrophy also supporting offline spike sorting, it differs from NeoAnalysis in several aspects: First, the data visualization in the SpikeSorting module of NeoAnalysis is developed based on the PyQtGraph [21], whereas the OpenElectrophy uses matplotlib [22]. Matplotlib is very slow and requires a vast amount of memory when plotting large amounts of data, thus is unsuitable for the visualization of large amounts of data. Second, the SpikeSorting module is more user-friendly. For example, it provides several easy-to-use widgets for the data selection process. In addition, the SpikeSorting module provides a 3D view to display the first three principal components. Data in this 3D space are plotted with appropriate transparency to reveal the data distribution density and users can interact with the 3D space using their mouse. In contrast, the 3D view provided by OpenElectrophy is unable to plot data in a transparent way and is not user-friendly.

### Future directions

In addition to the current functions, the NeoAnalysis toolbox can be expanded in the following (but not limited to) directions to meet the more specific demands of different users. First, in addition to the current spike sorting methods, we will try to provide more options for users if they are not satisfied with the current methods. Second, more plotting functions for both electrophysiological and behavioral data analyses, as well as more statistical options will be added. Plotting functions are packed as sub-functions of the graphics module, and, if users would like to, they are encouraged to include their own functions. Third, considering that there is no GUI for the graphics and the PopuAnalysis modules, we will develop a GUI for users who are not comfortable using scripts (such as simple commands). Fourth, for users who may encounter data import problems, we will offer to help users develop interfaces to import data of any format. In summary, we welcome users to interact with us to improve or modify the toolbox.

## Conclusions

In summary, NeoAnalysis is an open-source toolbox for electrophysiological data analysis. It provides many useful functions for general purposes, including the freely available module for offline spike sorting and other easy-to-use functions for plotting and analysis. We conclude that NeoAnalysis is a powerful toolbox for users doing electrophysiological experiments and is worth distributing in the field.

## Additional files

**Additional file 1.** Demo of spike detection. This movie illustrates how to use the SpikeDetection module to detect spikes from the sample data.

**Additional file 2.** Demo of spike sorting. This movie illustrates how to use the SpikeSorting module to perform offline spike sorting using the sample data.

## Abbreviations

PSTH: peristimulus time histogram
LFP: local field potential
I/O: input/output
GUI: graphic user interface
PCA: principal components analysis

## Footnotes

1 The NeoAnalysis v.1.0.0 can be freely downloaded from https://github.com/neoanalysis/NeoAnalysis.

2 For the purpose of stability and compatibility, NeoAnalysis integrates the Neo and PyQtGraph packages. Installation of these two packages is not necessary, however, users do need to install the other required packages, including *numpy*, *scipy*, *matplotlib*, *scikit-learn*, *quantities*, *pyopengl*, *pandas*, *h5py*, *statsmodels*, *PyWavelets*, and *seaborn*. The installation guide can be found in the user manual.
